# Improved Video-Based Point Cloud Compression via Segmentation

**DOI:** 10.3390/s24134285

**Published:** 2024-07-01

**Authors:** Faranak Tohidi, Manoranjan Paul, Anwaar Ulhaq, Subrata Chakraborty

**Affiliations:** 1 School of Computing Mathematics and Engineering, Charles Sturt University, Bathurst, NSW 2795, Australia; 2 School of Engineering and Technology, Centre for Intelligent Systems, Central Queensland University, Sydney Campus, Rockhampton, QLD 4701, Australia; a.anwaarulhaq@cqu.edu.au; 3 Faculty of Science, Agriculture, Business and Law, University of New England, Armidale, NSW 2351, Australia; subrata.chakraborty@une.edu.au; 4 Centre for Advanced Modelling and Geospatial Information Systems (CAMGIS), Faculty of Engineering and Information Technology, University of Technology Sydney, Ultimo, NSW 2007, Australia; 5 Griffith Business School, Griffith University, Brisbane, QLD 4111, Australia

**Keywords:** dynamic point cloud, compression, segmentation, V-PCC, 3D video

## Abstract

A point cloud is a representation of objects or scenes utilising unordered points comprising 3D positions and attributes. The ability of point clouds to mimic natural forms has gained significant attention from diverse applied fields, such as virtual reality and augmented reality. However, the point cloud, especially those representing dynamic scenes or objects in motion, must be compressed efficiently due to its huge data volume. The latest video-based point cloud compression (V-PCC) standard for dynamic point clouds divides the 3D point cloud into many patches using computationally expensive normal estimation, segmentation, and refinement. The patches are projected onto a 2D plane to apply existing video coding techniques. This process often results in losing proximity information and some original points. This loss induces artefacts that adversely affect user perception. The proposed method segments dynamic point clouds based on shape similarity and occlusion before patch generation. This segmentation strategy helps maintain the points’ proximity and retain more original points by exploiting the density and occlusion of the points. The experimental results establish that the proposed method significantly outperforms the V-PCC standard and other relevant methods regarding rate–distortion performance and subjective quality testing for both geometric and texture data of several benchmark video sequences.

## 1. Introduction

Recent advances in computer vision have made realistic digital representations of 3D objects and environmental surroundings possible. This allows real-time and realistic physical-world interactions for users [1,2,3], enabling real-world objects, people, and settings to move dynamically, applying 3D point clouds [4,5,6]. A point cloud is a set of individual 3D points without any order or relationship among them in the space. Each point has a geometry position and includes several other attributes such as transparency, reflectance, colour, and normal [7]. Dynamic point clouds are composed of a sequence of static three-dimensional point clouds, each representing a collection of sparsely sampled points taken from the continuous surfaces of objects and scenes. This unique structure serves as a powerful model for rendering realistic static and dynamic 3D objects [4,8,9,10]. The versatility of dynamic point clouds finds application in a broad spectrum of practical domains, encompassing geographic information systems, cultural heritage preservation, immersive telepresence, telehealth, and enhanced accessibility for individuals with disabilities. Furthermore, dynamic point clouds contribute to cutting-edge technologies such as 3D telepresence, telecommunication, autonomous driving, gaming, robotics, virtual reality (VR), and augmented reality (AR) [2,11]. Over the past decade, augmented and virtual reality have slowly entered the popular discourse and the Metaverse concept. The Metaverse is a virtual world that can create a network where anyone can interact through their avatars. An avatar can be a digital representation of a player and works as the identity of a natural physical person. If the Metaverse could be seamlessly connected with the physical environments in real time, it would transform our concept of reality [11]. Hence, the imperative lies in delivering a 3D virtual environment of the greatest quality, characterised by high resolution, minimal noise, and exceptional clarity, in order to achieve the highest degree of authenticity. Nevertheless, creating such high-fidelity 3D content demands a substantial allocation of resources for storage, transmission, processing, and visualisation. It is especially critical in sensor-based applications, where accurate and efficient 3D data processing is essential for the performance and trustworthiness of systems such as autonomous vehicles, robotics, and telehealth technologies [1,2,12].

Point clouds are categorised into three distinct groups, each with its designated standard and benchmark datasets to facilitate research comparisons. Category 1 pertains to static point clouds, exemplified by objects like statues and still scenes. Category 2 encompasses dynamic point clouds characterised by sequences involving human subjects. Lastly, Category 3 is reserved for dynamically acquired point clouds, a prime example being LiDAR point clouds [6,7,8,9]. Notably, recent advancements have given rise to two standardised approaches within the Moving Picture Experts Group (MPEG): video-based point cloud compression (V-PCC) and geometry-based point cloud compression (G-PCC) [1,2,4]. G-PCC, in particular, leverages data structures that excel in handling static scenarios, rendering it highly effective for addressing the requirements of both Category 1 and Category 3 of point clouds [13,14,15], while V-PCC exhibits superior performance in compressing the dynamic scenes, making it the more suitable choice for Category 2 [13,16,17]. In this study, the primary focus centres on Category 2, with the aim of enhancing V-PCC.

**Figure 1:**
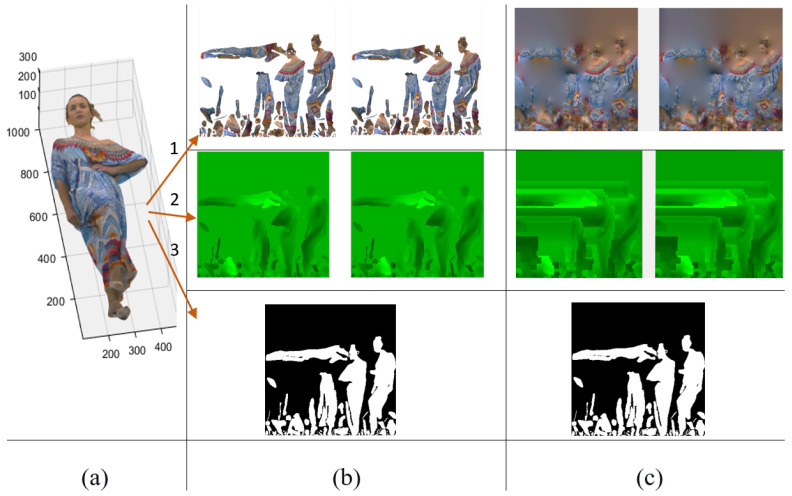
The process of patch generation and its three associated projected images by V-PCC. (**a**) Longdress point cloud; (**b**) three different associated projected images, including two layers of the texture map, two layers of geometry map, and occupancy map; (**c**) filled unused spaces between the patches in texture and geometry maps.

In the V-PCC method, illustrated in Figure 1, a point cloud is firstly divided into patches according to the normal directions of the points, then patches are projected and packed into three key mapping schemes, including texture, geometry, and occupancy. Figure 1a is one frame of a 3D point cloud named Longdress, and its three different associated images are shown in Figure 1b. The first and the second rows in Figure 1b include two layers of maps for geometry and texture. The last row shows an occupancy map of the point cloud. As seen from the images, V-PCC tries to place the data corresponding to each patch onto a 2D grid and minimise the space between the patches. Figure 1c shows a padding process aiming to fill unused spaces between the patches to make the generated texture and geometry frames more suitable for video coding. Figure 1c includes those three associated images with background filling for the texture and geometry.

However, there are challenges involved in the V-PCC method: (i) V-PCC loses the proximity of the patches as the neighbouring pixels can be included in different patches; (ii) V-PCC sacrifices some 3D points in order to have a limited number of 2D patches due to coding limitations; (iii) 2D video coding technique cannot compress the 2D projected images efficiently due to the larger amount of unoccupied space among the patches within the 2D images; (iv) 3D to 2D projection also introduces data loss as all self-occluded points cannot be retained due to the limited number of projection layers. All of these contribute to data loss and undesirable artefacts for inefficient coding and inferior visual quality [2,17]. Several compression methods have been proposed recently for point cloud processing; however, they still suffer from distortion issues [18]. The distortion occurs due to decreasing the number of the original points and changing the points’ position or colour, resulting in the degradation of the content quality. Tohidi et al. [19,20] manually divided a point cloud frame into smaller arbitrary-size patches and reported better performance of 2D video coding for some video sequences. However, without automatic segmentation and the consideration of occluded points [19,20], the effectiveness of these techniques in applications and performance is limited. Automatic segmentation significantly enhances overall performance and practicality in virtual and augmented reality applications.

The proposed method introduces a novel combination of cross-sectioning and slicing strategies to divide the entire point cloud into small sets of point clouds by considering the proximity of similar shapes and the number of self-occluded points. The cross-section strategy aims to increase the efficiency of 2D video coding by exploiting better spatial correlation, and the slicing strategy aims to reduce data loss by retaining more self-occluded points. Thus, these two approaches can reduce the overall artefacts, resulting in a better quality of the reconstructed point cloud. The cross-section divides the point cloud into several similar homogeneous cylindrical shapes to keep their proximity. Therefore, it helps provide patches of more regular shapes and sizes, which can fit together to reduce unused spaces among patches. At the same time, a 2D map is formed with patches to be encoded by the traditional 2D-based video coding techniques [21]. In the slicing approach, each cross-section is further sliced according to the number of self-occluded points in order to capture more original points and lessen data loss.

For further reduction of the data loss, overlapping slicing is considered. Furthermore, this paper includes the reconstruction of 3D point clouds and the impact of cross-section and slicing with analysis and comprehensive objective and subjective quality assessments with a wide range of point clouds. Therefore, the proposed method can provide greater temporal correlation with the assistance of these generated cross-sections. The experimental results demonstrate that the proposed method outperforms the V-PCC standard with a significant improvement in terms of rate–distortion (RD) performance in both texture and geometric point cloud compression. The results of the proposed method have also been compared with the cross-section [19] and slicing [20] approaches individually, showing that a proper combination of the approaches increases performance.

The key contributions of this paper are summarised below:

- We develop a method to automatically divide a point cloud into smaller segments by cross-sectioning based on coarse-level shape proximity. This approach enhances video coding by exploiting temporal and spatial redundancy through the reduction of the inter-patch spaces while forming 2D frames, i.e., Atlas.
- We further divide each cross-section into finer segments (i.e., slices) by innovatively determining the slicing direction within each cross-section based on self-occluded points so that it can reduce data loss for improving image quality.
- We introduce a variable slicing size based on the self-occluded points to capture more points in 2D projections for better image quality.
- Finally, we implement a technique to keep the slice sizes below a certain threshold to limit the required bits for the geometric positions of the slices.

A literature review is conducted in Section 2; the proposed method is outlined in Section 3; the proposed method’s results and discussion are presented in Section 4. The paper concludes with suggestions for future research directions in Section 5.

## 2. Literature Review

The first test models to compress point clouds were developed in 2017, and the available software for Category 2 was named TMC2 and for Categories 1 and 3, TMC13. MPEG approved the most recent standardisations, G-PCC and V-PCC, in 2020 and early 2021 [22,23]; however, there are certain limitations for both, demonstrating that there is still an opportunity for future advancement of current technology [24,25,26]. V-PCC coding relies on transforming 3D data to 2D data to take advantage of the existing video coding and being able to compress 3D data using 2D video coding. In contrast, G-PCC encodes the content directly in the 3D space. While V-PCC applies the existing 2D video compression approach to a collection of various 2D pictures transformed from the 3D point data, G-PCC applies Octree and K-D tree data structures to describe the placement of the points and their vicinities in the 3D space [27,28,29,30]. As V-PCC stands as the current state of the art in dynamic point cloud compression, this section introduces recent literature focused on enhancing the efficiency of V-PCC.

### 2.1. Enhancing V-PCC Efficiency through Improved 2D Video Coding

An input point cloud is broken down into a number of patches using V-PCC, which may subsequently be individually mapped and packed for video compression techniques like High-Efficiency Video Coding (HEVC) [21,31]. Researchers are experimenting with several techniques to increase video coding efficiency, including cuboid partitioning [32,33] and rate control [34,35]. Additionally, researchers have been working to enhance the patch production procedure and make it better suited for compressing transformed 3D data. These approaches include utilising edge detection for orienting motion [36], working on vacant pixels between patches [26,37] and applying 3D motion estimates [38,39]. Many studies integrated 2D and 3D information in the domain of bettering 2D map compression to improve RD performance. Finding matching blocks corresponding to content might be challenging for a 2D video encoder since packing has low consistency in neighbouring frames in 2D maps. The works by [38,39] seek to enhance the efficacy of motion estimation (ME) by merging 3D and 2D picture information in V-PCC. Thus, the scope of papers like [38] is constrained, limiting their applicability to enhance the efficiency of 2D video coding. The work outlined in [38] is particularly designed for low bitrates and demonstrates effectiveness primarily on specific datasets.

### 2.2. Enhancing V-PCC by Reducing Unused Spaces in 2D Maps

To enhance the V-PCC standard’s RD performance, Costa et al., in [40], suggested a novel patch-packing technique. Along with accompanying absolute and relative sorting and placement criteria, a number of unique patch-packing strategies were investigated. The RD performance improved in colour and geometry utilising the approach proposed by Costa et al. but could have improved in other domains. The accuracy of the 3D recreated item has been impacted because they altered the patch arrangement in the 2D maps [41]. Due to the inefficiency of coding idle areas during video compression, L. Li et al., in [42], have presented a method for reducing vacant pixels among various patches. An occupancy-map-based RD improvement that Li et al. gave increased compression efficiency, but required more RD performance. Since the TMC2 has problems creating a patch with numerous distinct contextual areas, which affects compression effectiveness, Rhyu et al., in [43], suggested a contextual homogeneity-based patch decomposing. Their approach eliminates the possibility of a single patch having many contextual regions in shape and colour. However, it is incompatible with extra properties like reflection and material ID. Another method that aims to diminish unused space in 2D maps introduced in [44] uses hexahedron segmentation. While this method enhances the efficiency of utilising 2D frames, it introduces seam-related issues among many hexahedrons in the reconstructed point cloud.

### 2.3. Enhancing Main View Quality in V-PCC

To increase the quality of the reconstructed point cloud, Zhu et al. [41] suggested allocating extra points to the patches associated with a predefined main view, by utilising the spots often dropped during patch production in order to optimise the visual experience for the user’s primary view. However, sacrificing other views can only improve the main view’s quality. A similar attempt has also been made in [45] to improve the main view for the user by saving more points for the main view patches. They could keep points from being seen from other views, improving the main view while boosting the compression bitrate. A method of dividing a whole point cloud into several portions has been presented in [19] to improve the effectiveness of 2D video coding and increase the quality of the main view. Partitioning is performed while considering the main view, shape, and size. Although it improves only the main view, some artefacts are still in the rebuilt point cloud, even in the main view, due to self-occluded points in concentrated areas.

### 2.4. Post-Processing to Eliminate Artefacts in V-PCC

The above-mentioned problems led to various artefacts in a V-PCC reconstructed point cloud, especially when high quantisation parameters (QPs) are used [46,47,48]. Cao and Cosman classified different geometric compression artefacts [49] and provided a detection and removal technique for each artefact. Jia et al.’s deep learning-based artefact removal is another proposal made in [50] in 2021. Several learning-based algorithms are also reviewed in the paper [51], most of which try to rectify the artefacts after appearance. However, this paper aims to decrease the likelihood of the artefacts appearing instead of rectifying them later, which can be more reasonable. The improved combination of the cross-section and slicing can decrease the artefacts and create the opportunity for parallel processing of the segments, resulting in more time effective.

### 2.5. Enhancing V-PCC Projection Layer Efficiency

V-PCC usually uses two layers of 2D projection for the whole point cloud to capture data. Fixed projection planes are considered ineffective for dynamic point cloud coding, according to the authors of [52]. Hence, a flexible technique is suggested with an adjustable projection plane number and orientation. Although single-layer mode in V-PCC has the benefit of fewer 2D maps requiring compression, it contains missing points. On the other hand, because the two-layer method needs extra bits, it could have a worse coding efficiency. As a result, various academics worked to improve the quality of single-layer mode encoding [53,54]. Sheikhipour et al. [54] suggested a technique for enhancing video coding efficiency and reconstruction quality by employing single-layer and patch-creation methods. This approach identified the distant layer’s most crucial areas and their involvement in the near layer’s patch creation. In other words, the technique of [54] tries to find the most critical patches of the far layer to include those in the near layer. As a result, this approach requires a reduced frame rate and memory buffers than the dual-layer procedure, making it appear more straightforward. In contrast, adding more data patches to the first layer enhances coding speed compared to merely considering one layer. However, a rebuilt point cloud’s geometric quality falls short of the V-PCC method. The approach in [43] recognises that many points may be overlooked due to the space between the inner and outer surfaces of the point cloud exceeding the limitation since the distance of the closest and farthest 3D points cannot surpass the predefined range boundary. The authors suggest creating a new patch to address the remaining points. They achieved bitrate savings of 0.5% on average. However, some points still need to be included to avoid appearing artefacts.

This paper aims to identify and mitigate factors causing artefacts in 3D reconstructed point clouds. Insufficient data are a crucial factor leading to artefacts, and this work addresses this by enhancing 2D video coding efficiency and collecting more data. Data are collected using only two layers of 2D projection; however, to enhance their performance, this paper concentrates these layers in areas with more self-occluded points. The proposed method, acting as a preprocessing step for V-PCC, offers the flexibility to incorporate additional improvement methods for further advancements. The proposed method has been benchmarked against similar approaches, including cross-section [19], slicing [20], motion estimation [38], and hexahedron segmentation [44], all aiming to enhance the efficiency of V-PCC. The proposed method consistently outperforms compared to the cross-section method [19] and achieves an average BD bitrate reduction of 6.3% and 6%, coupled with an average BD-PSNR improvement of 0.23 and 0.25 for geometry performance ($D_{1}$ and $D_{2}$). In texture performance, the proposed method achieves an average BD bitrate reduction of 7.4% and an average BD-PSNR improvement of 0.29 compared to the cross-section [19], a state-of-the-art approach in enhancing V-PCC.

## 3. Proposed Methodology

As the proposed method boosts V-PCC performance, this section begins by investigating the limitations of V-PCC. Subsequently, it outlines the strategies employed by the proposed method to address these limitations. In this section, the methodology of the proposed research is described.

### 3.1. Enhancing V-PCC Efficiency with the Proposed Method

V-PCC introduces three projection maps to include the point cloud dimensions: geometry, texture, and occupancy. A geometry map is created to embed depth values, and a texture map inserts the related attribute information, e.g., colour and light. An occupancy map is a binary image showing which location of the 2D maps is occupied and which is not. Then, all maps are compressed using existing video coding.

#### 3.1.1. Exploring Temporal Correlation in Generated Maps

**Figure 2:**
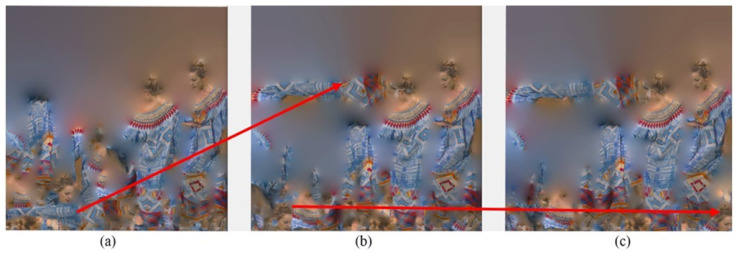
Poor temporal correlation among three consecutive texture frames of Longdress: (**a**) first frame, (**b**) second frame, and (**c**) third frame, where red lines indicate the blocks with the same content, but placed in different locations through the patch-generation strategy of the V-PCC standard.

The 2D maps in V-PCC have less temporal correlation than natural videos due to applying patch generation and packing, affecting the RD performance of video coding. In fact, with V-PCC, some blocks with the same content may be packed in different locations in sequential frames. This issue is illustrated in Figure 2. This figure demonstrates the poor temporal correlation of V-PCC using the first three frames of the Longdress video. Since frames obtained by V-PCC processing are not pure video, some strange behaviours may be seen. Like here, between three consecutive frames of Longdress, a big patch was indexed as another number in the second frame. Therefore, when the packetisation was performed, this patch jumped to a new position from its previous position. Similarly, several patches with the same content in the second and third frames have been packed in different locations. In Figure 2, red arrows point to these two jumps of the same contents. This kind of long jumping often happens in consecutive frames. It can cause challenges for the video decoder because video encoders usually look in the data’s neighbourhood to find the motion vectors. Due to this content inconsistency in the temporal direction, the video coding technique could not provide the best RD performance.

#### 3.1.2. Exploring the Causes of Data Loss

Points may be lost due to generating patches and projecting patches onto the 2D plane using V-PCC. Consequently, losing points or data loss may degrade the quality of a rebuilt point cloud and develop artefacts. Self-occlusions are the main reason for losing data when converting 3D data to 2D. Self-occlusions happen when more than one point has the same coordinate of dimension, so these points can be hidden by other points while projecting a 3D object onto 2D. Subsequently, the hidden points cannot be properly taken; therefore, these points will be missed after converting from 3D to 2D. The other reason for losing data is patch generation, causing data loss in three ways:

- Ignoring some isolated points.
- Losing points around each patch after compression.
- Projecting adjacent points onto different planes since these points are separated by patch generation.

**Figure 3:**
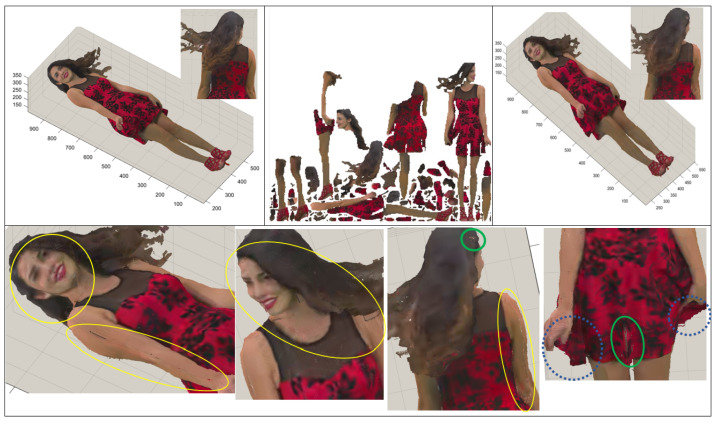
Different artefacts appear because of data loss while encoding by V-PCC, where a Redandblack video frame is used.

Figure 3 shows a point cloud named Redandblack compressed by V-PCC. Figure 3 (top) includes the original point cloud at the left-hand side, a 2D texture map in the middle, and a reconstrued point cloud on the right-hand side. In Figure 3, the bottom of the figure includes several portions of the reconstrued point cloud, zoomed in to display some artefacts clearly. These artefacts appeared on the reconstructed point cloud compressed by V-PCC. The images consist of artefacts marked by different-colour circles according to their types and reasons for appearance. Yellow circles show cracks occurring at the edge of patches. These sorts of artefacts are caused by dividing a point cloud into patches for 2D projection, disconnecting points that were once neighbours, and placing them at the margins of different patches. These nearby points might be projected onto different planes, and these 2D patches will undergo lossy compression. Thus, the patches’ margins may not align precisely with the reconstructed point cloud, causing the appearance of cracks. This can be proven by considering the related patches in the 2D texture map provided in Figure 3 (top-middle). Cracks in green circles are those points that are either self-occluded or ignored because they are isolated points. There is poor quality in blue dotted circles because only two layers of 2D projection are not enough since these regions are concentrated.

### 3.2. A Detailed Examination of the Proposed Method

The proposed approach involves a two-step preprocessing method for V-PCC, effectively tackling the specific challenges associated with V-PCC. These steps aim to improve the efficiency of 2D video coding and reduce the number of self-occluded points using cross-sectioning and slicing in order. Table 1 includes a summary of the equations’ symbols that have been used in the proposed method.

According to our final contribution, we implemented a technique to keep slice sizes within the predefined minimum and maximum thresholds, denoted as $\tau_{min}$ and $\tau_{max}$, respectively. The units for both $\tau_{max}$ and $\tau_{min}$ are the cubes of voxels used to represent the geometry position. For example, if the geometry position is represented using a 9-bit address space, the maximum and minimum sizes are calculated accordingly. Therefore, if 9 bits are allocated to represent the geometry position, the maximum size of each cross-section on any side cannot exceed 512 units (since $2^{9}=512$). This contrasts with the V-PCC method, which requires 10 bits for a point cloud size of 1024×1024×1024. It should be noted that, in some cases, the size of a slice and a cross-section can be the same; however, typically, the size of a slice is less than or equal to the size of a cross-section. Therefore, the total number of segments using the cross-section and slice must be true in the following equation.

(1) $$\kappa_{min}=L_{y}/\tau_{max}\;,\;\kappa_{max}=L_{y}/\tau_{min}$$

where $L_{y}$ is the length of the longest axis (assuming the y-axis is the longest axis), $\kappa_{min}$ is the least number of segments, and $\kappa_{max}$ is the most number of segments.

#### 3.2.1. The Initial Phase of the Proposed Method for Enhancing Temporal Correlation

The first issue is the inefficiency of video processing because of the irregular shapes of V-PCC patches, losing proximity, and having wasted space among them. The first step of the proposed method is to address this problem. The solution to this problem is cross-sectioning the whole point cloud so that different shapes in the point cloud can be separated into point cloud components that help maintain the proximity of the data points. When cross-sectioning, most parts are cut to achieve more regular shapes so they can be packed more closely, minimising the space between patches. As previously mentioned, a point cloud consists of a collection of coordinates (x, y, z) in three-dimensional space, representing the geometric positions of points. Point clouds are primarily defined by their surface rather than their volume, making it possible to identify similar shapes within them. Consequently, point clouds often contain multiple semicircular or semioval structures, forming various elliptical cylinders. To initiate the first step in this process, the point cloud needs to be cross-sectioned, with each cross-section containing points that belong to a similarly sized cylindrical shape. For points to be classified within the same cylindrical shape, the distance of each point from the centre of its corresponding segment should be approximately equal. The distance between the points on the rings or oval $\left(x_{i},z_{i},y\right)$ and their centres $\left(x_{c},z_{c},y\right)$ must be calculated to find the number of cylindrical segments. This distance (dy) can be calculated using the following equations (considering the *y*-axis is the longest axis and the value of y is constant in each ring):

(2) $$\left(x_{c},z_{c},y\right)=\left(\left(x_{max}-x_{min}\right)/2,\left(z_{max}-z_{min}\right)/2,y\right)$$

(3) $${\left(x_{i}-x_{c}\right)}^{2}+{\left(z_{i}-z_{c}\right)}^{2}=d_{y}^{2}.$$

Once the distances of the points situated on the surface of the point cloud from the centres are found, similar-sized cylindrical shapes can be achieved considering the definitions explained earlier as follows:

(4) $$\Delta d_{y}=\left|d_{y_{i}}-d_{y_{i+1}}\right|$$

**Figure 4:**
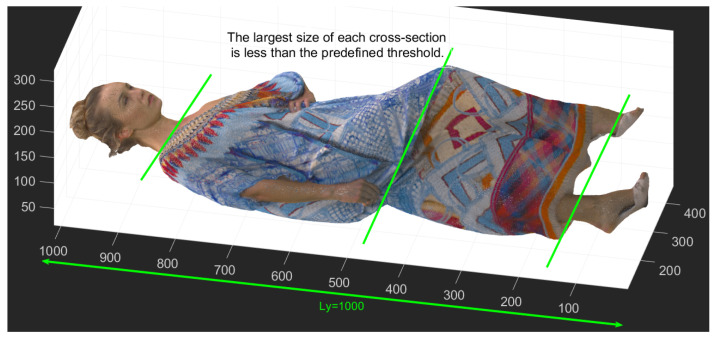
Example of four segments by the proposed cross-section process where the Longdress video sequence has been used.

$\Delta d_{y}$ is the absolute difference between $d_{y_{i}}$ and $d_{y_{i+1}}$ as defined in Equation (3). Here, $y_{i+1}$ are randomly chosen with the constraint that the distance between $d_{y_{i}}$ and $d_{y_{i+1}}$ is within the range of $\tau_{min}$ and $\tau_{max}$, where $d_{y_{1}}$ is set explicitly to $d_{Ymin}$ representing the minimum y-value in the point cloud. Once all $\Delta d_{y}$ are calculated, the number of “*k*” ($\kappa_{min}\leq k\leq \kappa_{max}$) of the greatest value of $\Delta d_{y}$ can be selected. Then, the point cloud is divided into *k* cross-sections from those maximums $\Delta d_{y}$. Next, any cross-section should be considered a new point cloud. Figure 4 shows that the Longdress point cloud has been cross-sectioned so that none of the cross-sections are bigger or smaller than the thresholds. The cross-sectioning Longdress produces more regular shapes with similar sizes that can be packed more closely, minimising the space between patches.

#### 3.2.2. The Second Phase of the Proposed Method for Reducing Data Loss

The second step addresses the second V-PCC issue mentioned before, data loss, by slicing each cross-section in a way that the number of self-occluded points decreases. On the one hand, slicing each cross-sectioned segment produced from the first step on its own can assist in including those points V-PCC might have ignored because of the restriction on the number of generated patches. On the other hand, in the second step, there is more attention on the areas that may lose data after projection. These regions would require increased maps for converting to 2D because they are highly at risk of self-occlusion. Self-occluded points mostly exist in the more concentrated areas or where there are changes in the normal direction of the points. These regions may need more than two layers of 2D maps for projection.

**Figure 5:**
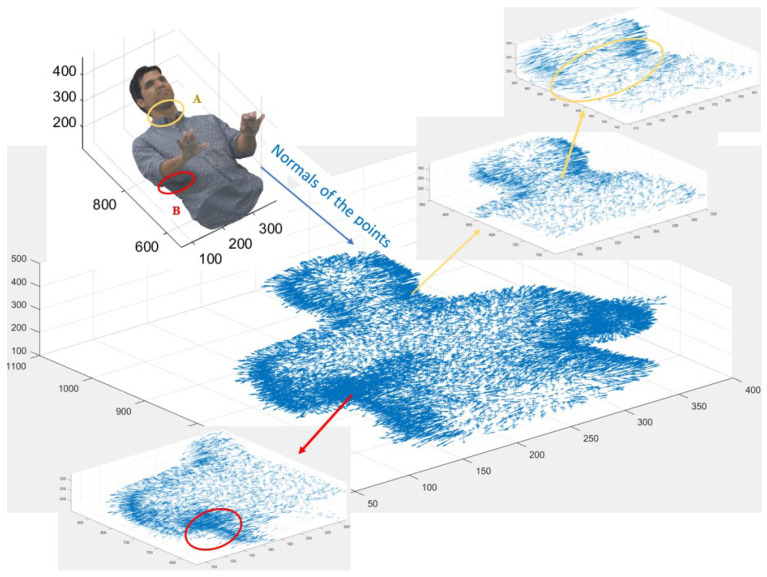
Normal estimation of Loot point cloud cross-sectioned and the areas (A, B) that risk data loss, where there is a change in the directions of the normals or a high density of the points.

Figure 5 shows a cross-section of the Loot point cloud and its points’ normals. Normal estimation is performed for the points available in this new point cloud (produced from step one), shown in blue at the bottom. Figure 5 illustrates areas that risk losing data after 2D projection, such as two spots in circles on the Loot. The normals’ image is enlarged twice on the right-hand side of Figure 5 and one time enlarged on the left-hand side. The first circle, shown in yellow and identified by “A”, includes the points with normals in different directions. Therefore, they need more planes with different angles for 2D projection; consequently, those points need to be sliced to project them into different planes, minimising data loss. Circle “B” on the point cloud in Figure 5, coloured red, is an example of the concentrated area, meaning that it includes more points per volume than the rest; therefore, extra layers of 2D projection might be needed in this position.

As mentioned before, V-PCC normally uses two layers of 2D projection, which is insufficient in concentrated regions. Therefore, slicing should be performed in these areas to capture more points. Since there are concentrated areas in any point cloud and some that are sparse, more layers of 2D projection will be needed in concentrated areas of the point cloud to capture all points. However, using slicing in the second step, the proposed method can handle covering more points without adding any extra layer. Slicing will be performed where the layers are needed, aiming to reduce the number of self-occluded points and increase the performance of the layers. Similar to step one, the sizes of all slices are between $\tau_{min}$ and $\tau_{max}$. The following procedures should be performed for any new point cloud produced from step one:

- Each side (direction) of the new point cloud (except the side already cross-sectioned in the first step) should be sliced from where the created 2D projection is the most.
  (5) $$\Omega_{S(i,j)}=\sum_{k=1}^{2}\alpha_{k(i,j)}\;,\;\left\{\begin{array}{cc} \; & 1\leq i\leq 5\\ \; & \tau_{min}\leq j\leq n/2 \end{array}\right.$$
  $\alpha_{k(i,j)}$ is the area of the 2D projection of the connected component of the $j_{th}$ slice in the $i_{th}$ side (direction). At most, the maximum number of sides the point cloud can be sliced from that direction can be five instead of six because one side has already been cross-sectioned using step one. It is assumed that the length of the point cloud in the $i_{th}$ side is *n*. From each side of the new point cloud, select a single, appropriate slice labelled as $S_{i}$ using conditions (5) and (6), aiming to generate the largest 2D projection data area in that direction.
  (6) $$Max\left(\Omega_{S(i,j)}\right)\Rightarrow S_{i}$$
- All selected slices will be compared in terms of the proportion of self-occluded points that can be calculated using the following formula:
  (7) $$\Psi_{Pi}=\Omega_{Si}/\Phi_{Pi}$$
  where $\Psi_{Pi}$ is the proportion of points captured successfully after 2D projection for the $i_{th}$ side. $S_{i}$ is the selected slide in the $i_{th}$ side and $\Omega_{Si}$ is the area of the 2D projection of $S_{i}$. $\Phi_{Pi}$ is the total number of points positioned in the selected slice of the $i_{th}$ side of the new point cloud. Thus, the most proportion value produced from (7) should be selected to find the best slice, and then, the rest can be considered a new point cloud, then slicing can be repeated to find the other best slice.

The repetitions can continue as long as the size of the slice is not less than $\tau_{min}$. After slicing, the whole number of self-occluded points can be obtained by the following formula:

(8) $$\varphi_{P}=\Phi_{P}-\sum_{k=1}^{m}\alpha_{k}$$

$\varphi_{P}$ is the number of self-occluded points in the new point cloud, and $\Phi_{P}$ is the total number of points in the new point cloud. $\alpha_{k}$ is the number of points captured successfully by the $k_{th}$ slice and can be achieved by calculating the areas of 2D projections of that slice, and “m” is the total number of created slices. In fact, the second step in this paper focuses on lessening the amount of $\varphi_{P}$ for each new point cloud created in step one.

**Figure 6:**
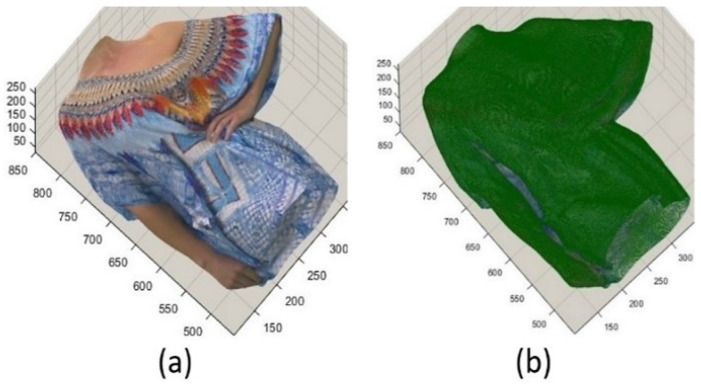
Cross-section created in step one of the proposed method and showing those points at risk of self-occlusion. (**a**) A Longdress’s cross-section; (**b**) green points can be captured accurately while the rest (mostly in blue) are at risk of self-occlusion.

**Figure 7:**
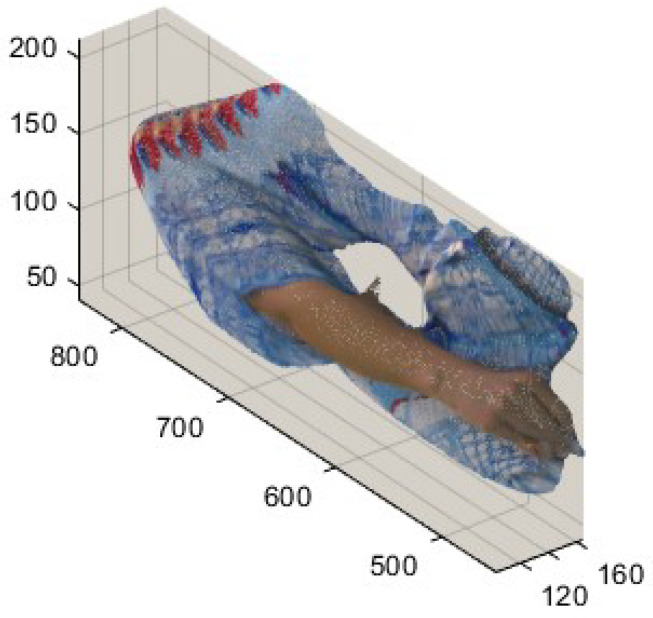
Creating a slice where the points are more at risk of self-occlusion to be able to capture more points (in the Longdress point cloud).

In the proposed method, overlapped slicing assists in keeping more original points and having more regular shapes of patches to be fit better in 2D maps. In addition, it can capture more points in concentrated regions. Overlapping is the repetition of solely the last line of each segment, encompassing the cross-section and slicing in the subsequent segment. Figure 6 and Figure 7 show how a cross-section produced by step one can be further sliced. Figure 6a displays a new point cloud, which is the biggest cross-section of Longdress shown in Figure 4. Figure 6b illustrates the points at risk of loss because of occlusion. The green points are the points that can be captured successfully using 2D projection, whereas the rest are self-occluded points. To be able to capture more points, a new point cloud will be sliced so that two layers of 2D projection can have their highest performance. Figure 7 displays the point clouds sliced from the concentrated regions where the risk of self-occluding could be high. This second step results in a considerable decrease in data loss and, consequently, decreases the incidence of artefacts in the rebuilt point cloud. The whole point cloud is segmented according to the two steps above, considering that each final segment size must not exceed a threshold in any direction. In this paper, the threshold for a maximum size of slicing is 512; therefore, the number of bits required to address each segment can be less than 10 (as usual), helping to decrease the required bitrate and compensate for the bitrate, which is supposed to be spent for overlapped regions. The rest is the same as V-PCC. Therefore, the proposed method is considered a preprocessing method to improve V-PCC performance. Since none of the proposed method’s cross-sectioning and the slicing steps estimate normals, the time consumed is tiny and incomparable to the time spent for patch generation in V-PCC.

## 4. Result and Discussion

The proposed performance is determined under common test conditions (CTCs) [22] using Test Model Category 2 (TMC2) to compare V-PCC and the performance of the proposed methods. We followed the common test condition of MPEG and JPEG standardisation of point cloud compression (PCC), which provides several representative dynamic point cloud sequences, including human beings’ point clouds, organised as full bodies and upper bodies. The 3D point cloud sequences defined by the MPEG PCC CTCs contain three categories [23], including type A, which contains the lowest complexity point cloud sequences (such as Loot, RedandBlack, and Soldier); type B (Longdress); and type C, which includes the highest complexity (Basketball Player). To ensure a comprehensive evaluation, we utilised various samples from different categories. The selected samples were drawn from sources such as 8i Voxelized Full Bodies (8i VFB) [55], Owlii [56], and the JPEG Pleno database, including Microsoft Voxelized Upper Bodies [57]. These diverse samples collectively contribute to a robust verification of the results, ensuring the reliability and relevance of our proposed approach in diverse scenarios and effectively allowing us to compare the performance of V-PCC and our proposed method. The proposed method has been benchmarked against similar approaches, including the cross-section [19], slicing [20], motion estimation [38], and hexahedron segmentation [44], all aiming to enhance the efficiency of V-PCC.

The proposed method and Kim et al.’s method [38] aim to improve V-PCC’s efficiency by reducing projection errors. Therefore, Kim et al.’s method [38] is included in the comparison section in addition to V-PCC. It [38] (motion estimation) employs a 3D motion estimation method to generate a reference point cloud frame, utilising it to enhance coding by mitigating projection errors. In contrast, the proposed method reduces error by segmenting point clouds into smaller parts.

Similarly, the cross-section [19] and slicing [20] employ manual division, focusing on similar shapes and occlusion. Furthermore, hexahedron segmentation [44] utilises hexahedrons to segment a point cloud and boost video coding efficiency within V-PCC. The results have been achieved after applying both steps of the proposed method, including several visual and objective comparisons.

This paper sets $\tau_{max}$ to 512 units and $\tau_{min}$ to 32 units, requiring 9 bits to address the geometry, which is less than the 10 bits required by the standard V-PCC. While adjusting $\tau_{max}$ to a smaller size can improve quality, it also increases segmentation time. Similarly, setting $\tau_{min}$ too small can enhance quality by reducing self-occluded points, but it also raises the bitrate and processing time. There is a trade-off between the threshold sizes and the resulting bitrate and time complexity. This trade-off must be balanced according to the specific requirements of the application.

**Figure 8:**
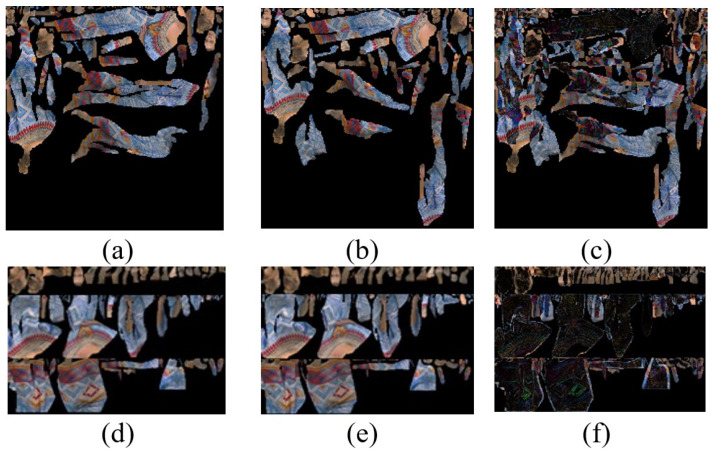
Two consecutive texture frames of Longdress to demonstrate improved temporal correlation and reduced wasted space by the proposed method compared with V-PCC. (**a**) V-PCC first frame, size: 1280 × 1320, unused space: 64%. (**b**) V-PCC second frame, size: 1280 × 1320. (**c**) Difference between these two frames: 50%. (**d**) The proposed method first frame, size = 1280 × 896, unused space: 47%. (**e**) The proposed method second frame size = 1280 × 896. (**f**) Difference between these two frames: 42%.

**Figure 9:**
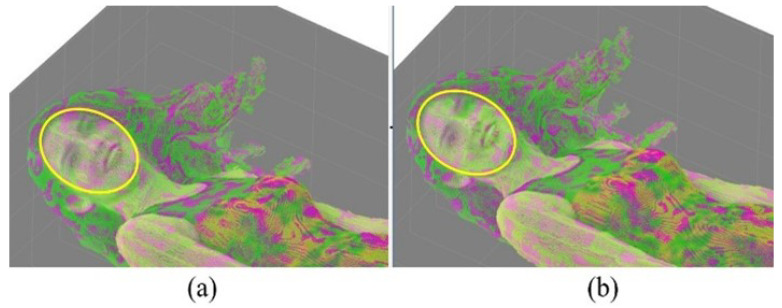
Comparison of data loss, (original data are in green, and the rest are artificial). (**a**) V-PCC and (**b**) the proposed method, demonstrating that the proposed method includes more original data.

**Figure 10:**
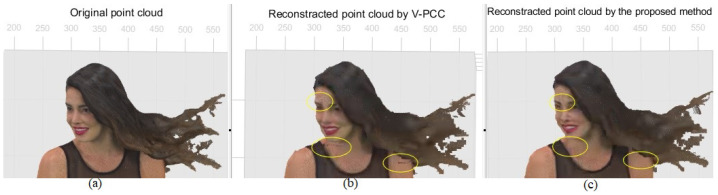
Improving the cracks around the face’s patch, marked by the yellow colour in Figure 3. (**a**) Original point cloud; (**b**) reconstructed by V-PCC; (**c**) reconstructed by the proposed method.

**Figure 11:**
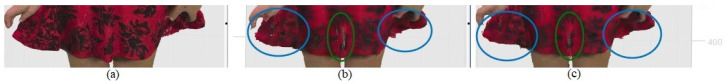
Improving the artefacts marked by the green and blue dotted colours in Figure 3. (**a**) Original point cloud. (**b**) Reconstructed by V-PCC. (**c**) Reconstructed by the proposed method.

Several visual comparisons are provided and displayed in Figure 8, Figure 9, Figure 10 and Figure 11.

Figure 8 shows two consecutive texture frames from the ‘Longdress’ sequence presented in Figure 8a,b, aiming to illustrate the enhanced temporal correlation and reduced unused space achieved by the proposed method using the same frames shown in Figure 8d,e. The visual comparison within the figure offers a qualitative assessment of the improved time correlation resulting from the proposed method. This improvement can be seen in the third column of the figure, so the difference between two consecutive frames using V-PCC is 50% (Figure 8c). In contrast, this difference by the proposed method decreased to 42% (see Figure 8f), proving improved temporal correlation by the proposed method. The reason is that, with V-PCC, some blocks with the same content may be packed in different locations in sequential frames, as illustrated in Figure 2. After packing all patches in a 2D map, some patches may jump to a new position from their previous position because there is not enough room in the same place. However, this kind of long jumping may not happen in consecutive frames when the cross-sectioning preprocessing has been performed. With cross-sectioning, similar body parts will be arranged in the same or neighbouring areas in the 2D map. Moreover, Figure 8 displays that the proposed method, unlike V-PCC, generates patches with enhanced regularity in shape, featuring at least one straight-line side and relatively modest in size. This characteristic facilitates more efficient packing of the patches. Therefore, the patches can fit together more closely, decreasing unused space among patches. Reducing unused space in the frames is also visually supported by Figure 8. The unused space is reduced from 64% with V-PCC to 47% with the proposed method. Consequently, the proposed method achieves a smaller frame size of 1280 × 896, in contrast to V-PCC’s frame size of 1280 × 1320.

Figure 9 shows the reconstructed point cloud of Redandblack by V-PCC in Figure 9a and the proposed method in Figure 9b. Figure 9 includes a comparison with the original point cloud so that those points that are the same as the original points are marked in green, and the rest are in pink. A yellow circle on the face of the Redandblack point cloud marks the comparison area. More green points in Figure 9b than in Figure 9a mean that the proposed method has kept more original data than V-PCC, e.g., in the face.

A comparison can be made using Figure 10, especially where artefacts appeared, e.g., for those marked in Figure 3, proving the proposed method improved the quality of the rebuilt point cloud. Figure 10 demonstrates those noticeable cracks on the face, neck, and shoulder of Redandblack using V-PCC, as shown in Figure 3 (marked by yellow circles), rectified using the proposed method. Using V-PCC, data are lost at the edges of patches; furthermore, patches for the face are projected onto different planes, losing their connection and proximity. In this case, applying overlapping slices decreases data loss, especially at the edge of the patch, resulting in amending the cracks. Figure 11 shows other artefacts explained in Figure 3 (marked by green and blue dotted circles), which still need rectification even using the proposed method. However, the proposed method’s quality of the reconstructed point cloud is better than that of V-PCC because it considers those points that may be self-occluded by V-PCC. Table 2 compares the proportion of data loss between V-PCC and the proposed methods across various point cloud sequences. The results highlight the efficacy of the proposed methods in preserving a higher proportion of original points compared to V-PCC.

**Figure 12:**
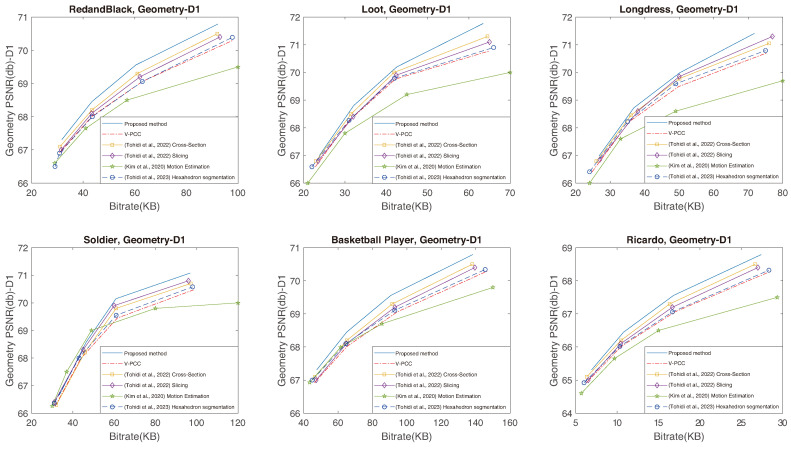
Geometry ($D_{1}$) RD curves using the proposed method and the V-PCC, cross-section [19], slicing [20], motion estimation [38], and hexahedron segmentation [44] methods for various standard video sequences.

**Figure 13:**
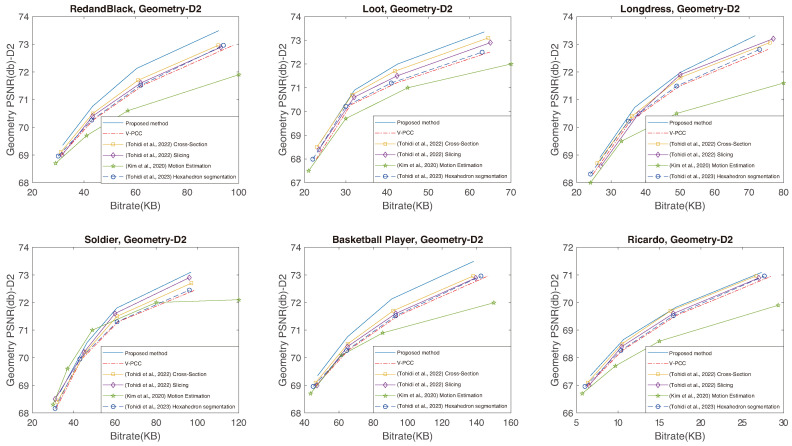
Geometry ($D_{2}$) RD curves using the proposed method and the V-PCC, cross-section [19], slicing [20], motion estimation [38], and hexahedron segmentation [44] methods for various standard video sequences.

**Figure 14:**
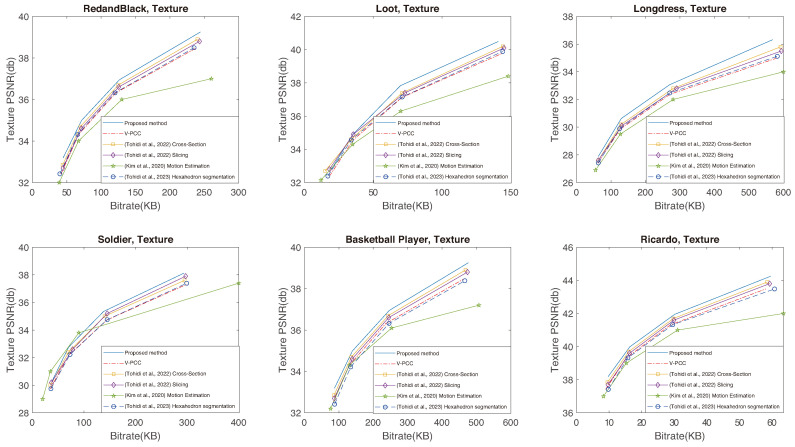
Texture (YUV) RD curves using the proposed method and the V-PCC, cross-section [19], slicing [20], motion estimation [38], and hexahedron segmentation [44] methods for various standard video sequences.

The capacity of the proposed method to establish increased temporal correlation between frames, coupled with its ability to preserve a larger volume of original data, leads to superior performance. This means an enhancement in the quality of the reconstructed point cloud, achieved with a reduced bitrate when compared to V-PCC, as clearly illustrated in Figure 12, Figure 13 and Figure 14. The proposed method excels in creating RD curves compared to the existing methods by strategically employing the cross-section for improved temporal correlation and preserving more original points through slicing. Figure 12 and Figure 13 represent the geometry RD curves, evaluating the proposed method against the V-PCC, cross-section [19], slicing [20], motion estimation [38], and hexahedron segmentation [44] methods for various standard video sequences, explicitly focusing on point-to-point ($D_{1}$) and point-to-plane ($D_{2}$) distance metrics. These metrics assess the geometric quality of a reconstructed point cloud by measuring the peak signal-to-noise ratio (PSNR) to calculate the average distances between each point in the original cloud and its nearest neighbour in the decompressed cloud. Specifically, $D_{1}$ relies on point-to-point distances, while $D_{2}$ incorporates point-to-plane distances. Additionally, Figure 14 illustrates texture (YUV) RD curves for the same methods and video sequences. The corresponding BD bitrate and BD-PSNR values can be found in Table 3, Table 4 and Table 5, aligning with six standard video sequences introduced earlier to complement the graphical representation. The BD bitrate, or Bjontegaard Delta Rate, is a valuable metric for comparing bitrates between video codecs or coding configurations, offering insights into their RD performance difference. Negative values indicate a reduction, while positive values signify an increase in the bitrate.

The RD curves shown in Figure 12, Figure 13 and Figure 14 prove that the proposed method can perform better than the other methods, except for Soldier at a lower bitrate, for which Kim et al.’s method [38] performs better. There are two reasons for this exception: firstly, the Soldier sequence includes many irregular shapes that can hardly be divided using cross-sectioning, and secondly, the 3D motion between frames in the Soldier sequence is very small. Kim et al.’s method [38] can find them better using its 3D motion search. The results presented in the tables emphasise a substantial improvement in both the BD-PSNR and BD bitrates with the proposed method. The suggested approach demonstrates the ability to reconstruct a higher quality point cloud even when operating at a lower bitrate. These comparative analyses reinforce the viability and superiority of our proposed method, establishing it as a robust solution for efficient point cloud compression with improved geometry and texture preservation. To elaborate on the determination of thresholds $\tau_{max}$ and $\tau_{min}$, these values were selected to be 512 and 32 considering the dimension size of the point cloud, specifically the size of the smallest and largest sides of the point cloud. Altering these values can impact performance; reducing the sizes increases processing time, while increasing the size can lead to a performance decline.

The proposed method exhibits flexibility in the number and size of cross-sections and slices, intelligently adapting to the proximity of shapes and self-occluded points. This dynamic adjustment enhances temporal correlation and effectively mitigates data loss. Moreover, since the proposed method acts as a preprocessing step for V-PCC, it may also offer the flexibility to incorporate additional pre-processing methods for further advancements. The time spent on the proposed method is not much, thanks to not using normal estimation and refining processes to generate cross-sections or slices. The proposed method requires an additional time of approximately 8.86% compared to only the patch generation phase in the V-PCC process. However, the proposed approach facilitates parallel processing by generating independent segments, substantially reducing patch-generation time. Moreover, if we consider the extra time requirement against the whole V-PCC encoding process, it is only around 4% as patch generation takes less than 50% of the encoding time. Table 6 provides the requirement of the time by the proposed preprocessing method to create segments by the cross-section and slicing steps using different video sequences. The comparison criterion is based on the time required for V-PCC coding.

## 5. Conclusions

The focus of this paper is to propose a method to address the limitations of the existing standard of dynamic point cloud compression (V-PCC), including increasing the efficiency of 2D video coding and decreasing data loss. The most concerning reasons for V-PCC problems are patch production and the subsequent conversion of 3D patches to 2D, creating artefacts on the rebuilt point cloud. Therefore, the proposed method focuses on these areas to deliver greater temporal correlation and reduce data loss while captured data are being processed. This goal has been achieved using two segmentation steps by the proposed method providing similar shapes with decreased self-occluded points to overcome the V-PCC issues. Each segment produced while applying two segmentation steps is considered with one line overlapped to rectify data loss at the edge of the segment.

The proposed method consistently outperforms the standard V-PCC, as well as recent advancements represented by the cross-section [19], slicing [20], motion estimation [38], and hexahedron segmentation [44] methods across diverse point cloud sequences. Notable enhancements include an average BD bitrate reduction of 6.3% and 6%, coupled with an average BD-PSNR improvement of 0.23 and 0.25 for geometry performance ($D_{1}$ and $D_{2}$), surpassing the state-of-the-art cross-section method [19], which currently stands as the most effective in improving V-PCC’s performance. Regarding texture performance, the proposed method achieves an average BD bitrate reduction of 7.4% and an average BD-PSNR improvement of 0.29 when compared to the cross-section [19], a state-of-the-art approach for enhancing V-PCC. These findings underscore the effectiveness of our approach in preserving both geometry and texture, positioning it as a robust solution for efficient point cloud compression. The demonstrated ability to provide a more accurate representation of the original point clouds further solidifies the efficacy of our proposed method.

In the initial phase of the proposed method, the point cloud is segmented based on similar shapes without accounting for the sparsity and density of that region. Future improvements could focus on incorporating considerations for the density and sparsity of the segmented region during the cross-section process.

## Figures and Tables

**Table 1:** Summary of the equations’ symbols.

Symbol	Description
$\tau_{min}$ and $\tau_{max}$	Minimum and maximum size of segments
$L_{y}$	Length of the longest axis
$\kappa_{min}$ and $\kappa_{max}$	Minimum and maximum number of segments
$d_{y}$	Distance of two points when the value of y is constant
Ω	The total area of 2D projection
α	The area of 2D projection of a slice
$S_{i}$	The selected slide in the ith side
φ	The number of self-occluded points
Ψ	The proportion of points captured successfully
Φ	Total number of points

**Table 2:** Comparison of the proportion of data loss using V-PCC and the proposed method.

Sequence	V-PCC	Proposed Method
Redandblack	9.4%	7.9%
Loot	12%	10.4%
Longdress	9.8%	8.1%
Soldier	11%	10.1%
Basketball Player	10.7%	9.9%
Ricardo	9.6%	9.5%
Average	**10.4%**	**9.3%**

**Table 3:** BD bitrate and BD-PSNR of geometry performance (point to point = $D_{1}$) of the proposed method against the V-PCC, cross-section [19], slicing [20], motion estimation [38], and hexahedron segmentation [44] methods for various point cloud sequences.

Sequence	BD Bitrate					BD-PSNR (dB)				
	V-PCC	[19] Cross-Section	[20] Slicing	[38] Motion Estimation	[44] Hexahedron Segmentation	V-PCC	[19] Cross-Section	[20] Slicing	[38] Motion Estimation	[44] Hexahedron Segmentation
Redandblack	−15.8%	−7.8%	−11.4%	−24.0%	−15.8%	0.53	0.26	0.39	0.85	0.54
Loot	−8.8%	−4.2%	−7.4%	−18.4%	−8.0%	0.45	0.18	0.35	1.04	0.39
Longdress	−9.7%	−5.0%	−5.1%	−23.4%	−7.3%	0.44	0.21	0.20	1.1	0.32
Soldier	−9.4%	−6.6%	−2.8%	−1.2%	−6.7%	0.54	0.32	0.15	0.45	0.41
Basketball Player	−15.8%	−7.8%	−11.4%	−17.0%	−12.9%	0.53	0.26	0.39	0.61	0.42
Ricardo	−16.9%	−6.5%	−11.3%	−27.5%	−15.3%	0.44	0.17	0.30	0.76	0.40
Average	**−12.7%**	**−6.3%**	**−8.2%**	**−18.6%**	**−11.0%**	**0.49**	**0.23**	**0.30**	**0.80**	**0.41**

**Table 4:** BD bitrate and BD-PSNR of geometry performance (point to plane = $D_{2}$) of the proposed method against the V-PCC, cross-section [19], slicing [20], motion estimation [38], and hexahedron segmentation [44] methods for various point cloud sequences.

Sequence	BD Bitrate					BD-PSNR (dB)				
	V-PCC	[19] Cross-Section	[20] Slicing	[38] Motion Estimation	[44] Hexahedron Segmentation	V-PCC	[19] Cross-Section	[20] Slicing	[38] Motion Estimation	[44] Hexahedron Segmentation
Redandblack	−15.4%	−9.1%	−12.1%	−27.8%	−13.8%	0.62	0.38	0.51	1.18	0.56
Loot	−12.1%	−3.2%	−7.2%	−21.2%	−9.9%	0.67	0.18	0.41	1.03	0.55
Longdress	−9.2%	−5.0%	−6.1%	−24.9%	−7.5%	0.41	0.20	0.23	1.18	0.34
Soldier	−10.1%	−6.9%	−3.3%	−0.3%	−8.5%	0.48	0.29	0.13	0.18	0.42
Basketball Player	−15.3%	−9.1%	−12.1%	−20.1%	−13.9%	0.62	0.38	0.51	0.92	0.56
Ricardo	−11.2%	−2.7%	−7.0%	−30.4%	−9.3%	0.31	0.07	0.20	0.86	0.25
Average	**−12.2%**	**−6.0%**	**−7.9%**	**−20.8%**	**−10.5%**	**0.52**	**0.25**	**0.33**	**0.89**	**0.45**

**Table 5:** BD bitrate and BD-PSNR of texture performance of the proposed method against the V-PCC, cross-section [19], slicing [20], motion estimation [38], and hexahedron segmentation [44] methods for various point cloud sequences.

Sequence	BD Bitrate					BD-PSNR (dB)				
	V-PCC	[19] Cross-Section	[20] Slicing	[38] Motion Estimation	[44] Hexahedron Segmentation	V-PCC	[19] Cross-Section	[20] Slicing	[38] Motion Estimation	[44] Hexahedron Segmentation
Redandblack	−13.3%	−6.1%	−9.6%	−24.6%	−11.7%	0.51	0.23	0.37	1.08	0.44
Loot	−13.9%	−6.0%	−7.5%	−24.2%	−11.8%	0.57	0.29	0.30	1.12	0.48
Longdress	−18.4%	−10.6%	−14.0%	−29.0%	−16.4%	0.75	0.43	0.57	1.22	0.67
Soldier	−16.5%	−9.0%	−9.1%	−2.4%	−17.0%	0.67	0.36	0.37	0.15	0.69
Basketball Player	−13.3%	−6.1%	−9.6%	−20.6%	−16.8%	0.51	0.23	0.36	0.89	0.66
Ricardo	−14.2%	−6.6%	−10.3%	−26.1%	−16.6%	0.50	0.22	0.37	1.06	0.62
Average	**−14.9%**	**−7.4%**	**−10.0%**	**−21.1%**	**−15.0%**	**0.58**	**0.29**	**0.39**	**0.92**	**0.59**

**Table 6:** Extra time required for combined cross-section and slicing using the proposed methods versus the V-PCC patch generation process.

Sequence	Step 1 Cross-Section	Step2 Slicing	Total Extra Time
Redandblack	2.90%	6.50%	9.40%
Loot	2.50%	5.20%	7.70%
Longdress	3.20%	6.60%	9.80%
Soldier	3.45%	6.55%	10.00%
Basketball Player	2.65%	5.50%	8.15%
Ricardo	2.15%	5.95%	8.10%
Average	**2.81%**	**6.05%**	**8.86%**

## Data Availability

All data generated or analysed during this study are included in this published article.
